# Characterization and Genome Analysis of the First Facultatively Alkaliphilic *Thermodesulfovibrio* Isolated from the Deep Terrestrial Subsurface

**DOI:** 10.3389/fmicb.2016.02000

**Published:** 2016-12-19

**Authors:** Yulia A. Frank, Vitaly V. Kadnikov, Anastasia P. Lukina, David Banks, Alexey V. Beletsky, Andrey V. Mardanov, Elena I. Sen’kina, Marat R. Avakyan, Olga V. Karnachuk, Nikolai V. Ravin

**Affiliations:** ^1^Laboratory of Biochemistry and Molecular Biology, Tomsk State UniversityTomsk, Russia; ^2^Institute of Bioengineering, Research Center of Biotechnology of the Russian Academy of SciencesMoscow, Russia; ^3^Systems, Power and Energy, School of Engineering, Glasgow UniversityGlasgow, UK; ^4^Holymoor Consultancy Ltd.Chesterfield, UK

**Keywords:** alkaliphilic bacteria, deep subsurface biosphere, genome analysis, sulfate reduction, *Thermodesulfovibrio*

## Abstract

Members of the genus *Thermodesulfovibrio* belong to the *Nitrospirae* phylum and all isolates characterized to date are neutrophiles. They have been isolated from terrestrial hot springs and thermophilic methanogenic anaerobic sludges. Their molecular signatures have, however, also been detected in deep subsurface. The purpose of this study was to characterize and analyze the genome of a newly isolated, facultatively alkaliphilic *Thermodesulfovibrio* from a 2 km deep aquifer system in Western Siberia, Russia. The new isolate, designated N1, grows optimally at pH 8.5 and at 65°C. It is able to reduce sulfate, thiosulfate or sulfite with a limited range of electron donors, such as formate, pyruvate, and lactate. Analysis of the 1.93 Mb draft genome of strain N1 revealed that it contains a set of genes for dissimilatory sulfate reduction, including sulfate adenyltransferase, adenosine-5′-phosphosulfate reductase AprAB, membrane-bound electron transfer complex QmoABC, dissimilatory sulfite reductase DsrABC, and sulfite reductase-associated electron transfer complex DsrMKJOP. Hydrogen turnover is enabled by soluble cytoplasmic, membrane-linked, and soluble periplasmic hydrogenases. The use of thiosulfate as an electron acceptor is enabled by a membrane-linked molybdopterin oxidoreductase. The N1 requirement for organic carbon sources corresponds to the lack of the autotrophic C1-fixation pathways. Comparative analysis of the genomes of *Thermodesulfovibrio* (*T. yellowstonii, T. islandicus, T. àggregans, T. thiophilus*, and strain N1) revealed a low overall genetic diversity and several adaptive traits. Consistent with an alkaliphilic lifestyle, a multisubunit Na^+^/H^+^ antiporter of the Mnh family is encoded in the *Thermodesulfovibrio* strain N1 genome. Nitrogenase genes were found in *T. yellowstonii, T. aggregans*, and *T. islandicus*, nitrate reductase in *T. islandicus*, and cellulose synthetase in *T. aggregans* and strain N1. Overall, our results provide genomic insights into metabolism of the *Thermodesulfovibrio* lineage in microbial communities of the deep subsurface biosphere.

## Introduction

The genus *Thermodesulfovibrio* was established in 1994 following the isolation of a novel thermophile, *Thermodesulfovibrio yellowstonii*, from hydrothermal vent in Yellowstone Lake (Henry et al., 1994). Two other species, *T. islandicus* (Sonne-Hansen and Ahring, 1999) and *T. hydrogeniphilus* (Haouari et al., 2008), were subsequently isolated from terrestrial hot springs in Iceland and Tunisia, respectively. Two of the five described species, *T. aggregans* and *T. thiophilus*, were isolated from thermophilic methanogenic sludge samples (Sekiguchi et al., 2008). To date, none of the cultivated *Thermodesulfovibrio* has been isolated from the deep subsurface. Molecular signatures of this group have, however, been detected in deep aquifer systems of the Great Artesian Basin, Australia (Kimura et al., 2005), in formation waters and oil from several reservoirs in China (Nazina et al., 2006; Wang et al., 2014; Yang et al., 2016) and in deep granitic groundwater from the Grimsel Test Site, Switzerland (Konno et al., 2013).

The genus *Thermodesulfovibrio* is assigned to the *Nitrospirae* phylum, where it forms a separate deep-branching group, distinct from the sister clades *Nitrospira* and *Leptospirillum* (Daims, 2014). Kinusawa (2010) showed a close evolutionary relationship between *T. yellowstonii* and *Proteobacteria* by the use of gene order data from completely sequenced genomes.

All known *Thermodesulfovibrio* isolates are strict anaerobes and use a limited number of organic electron donors, which are incompletely oxidized to acetate. They all are capable of dissimilatory reduction of sulfate and thiosulfate, and some species can also use sulfite, Fe(III)-NTA, and nitrate as electron acceptors. Molecular hydrogen can be used as an energy source. The temperature range for growth is from 40 to 75°C. The upper pH limit for growth is 7.7–8.5 for all species. The lower pH limit varies from 6.0 (*T. aggregans, T. thiophilus*, and *T. islandicus*) to 6.5 (*T. yellowstonii* and *T. hydrogeniphilus*). Acidophilic or alkaliphilic representatives of the genus have not been described.

The complete genome of *T. yellowstonii* DSM 11347^T^ (Bhatnagar et al., 2015) and a draft genome of *T. aggregans* TGE-P1^T^ (Matsuura et al., 2016) have been published. The draft genomes of *T*. *islandicus* DSM 12570 (NZ_AXWU01000000) and *T*. *thiophilus* (NZ_AUIU01000000) are also available in GenBank. To date, none of these genomes has been analyzed in detail.

Here, we report the isolation and characterization of the first moderately alkaliphilic *Thermodesulfovibrio* from a 2 km deep subsurface aquifer system. The comparative genome analysis of this isolate and the four known species provides information on *Thermodesulfovibrio* life styles and metabolism.

## Materials and Methods

### Sampling Site, Field Measurements, and Chemical Analyses

Groundwater was sampled from a deep artesian borehole, drilled in 1961–1962 for the purpose of oil exploration. The borehole is designated 1-R and is located in the town of Byelii Yar in the Tomsk region of Western Siberia (58.4496°N 85.0279°E; Supplementary Figure S1). No significant hydrocarbon reserves were discovered in the borehole and today it overflows with warm (40–45°C), slightly brackish (ca. 1.8 g L^-1^ mineralisation and 830–840 mg L^-1^ chloride) groundwater, which is used for small scale commercial and domestic purposes. The borehole is located in a small building on the south bank of River Ket’ (a tributary of River Ob’), about 150 m from the river and at a ground elevation of 84 m asl. The borehole was originally drilled to 2.56 km deep: it passed through Quaternary and Tertiary sediments to 170 m and thereafter a thick sequence of Danian, Cretaceous and Jurassic sedimentary deposits until it entered Palaeozoic basement at 2,505 m (comprising sedimentary rocks to 2,534 m and thereafter basalts to 2,563 m).

Following standard oil industry practice at the time, the borehole was tested using the bottom-up casing perforation method, whereby successive sections of casing were perforated and pressure/flow tested. After testing, the section was sealed by cement and a new, higher section perforated. The last section to be tested was the interval from 1997 to 2005 m in the Hauterivian-Barremian (i.e., early Cretaceous) sedimentary rocks of the Ilekskaya Suite (with a cement seal having been emplaced up to 2,172 m depth). It is thus this interval of the aquifer system which is assumed to be yielding today’s artesian groundwater flow (and the depth is consistent with a typical continental geothermal gradient of ca. 2°C per 100 m and a groundwater temperature of 40–45°C; Banks, 2012).

According to the ^18^O and ^2^H stable isotope composition analyzed previously (Banks et al., 2014), the water is essentially derived from meteoric recharge, with the isotopic signature slightly modified by ^18^O exchange with the aquifer matrix in a mildly geothermal environment or possibly by admixture with a minor component of connate marine water.

Sampling and field measurements were taken on August 23–24, 2013. Water temperature, pH and Eh were measured at the wellhead using pH-meter HI 8314 (Hanna Instruments Deutschland, Vöhringen) with appropriate electrodes. The water sample was fixed with 2.4% Zn-acetate in proportion of 1:5 for hydrogen sulfide determination. H_2_S was measured colorimetrically with the methylene blue method (Cline, 1969) in triplicate using a Smart Spec Plus spectrophotometer (Bio-Rad Laboratories, Hercules, CA, USA).

### Enrichment and Pure Culture Isolation

The initial enrichment was set up in liquid freshwater Widdel and Bak (1992) (WB) medium that contained (per liter) 4 g Na_2_SO_4_, 0.2 g KH_2_PO_4_, 0.25 g NH_4_Cl, 1 g NaCl, 0.4 g MgCl_2_⋅6H_2_O, 0.5 g KCl, 0.113 g CaCl_2_, 2 ml of vitamin solution, 1 ml of microelement solution, 1 ml each of Na_2_SeO_3_ (final concentration 23.6 μM), and Na_2_WO_4_ (24.2 μM) solutions (Widdel and Bak, 1992). Medium was adjusted to pH 7.5 with NaHCO_3_ and Na_2_S⋅9H_2_O was used as a reducing agent. Each cultivation vial received an iron wire (100% Fe) as previously described (Karnachuk et al., 2008) as an additional micro source of iron. Gelatin (1%), citrate (0.1%), pyruvate (0.1%), formate (0.1%), and peptone (1%) were supplied as electron donors with 28 mM sulfate as an electron acceptor. Several 12 mL vials with sterile medium and 3 mm glass beads (1/4 of the vial volume) were inoculated on-site with 2.5 mL water sample per each vial by sterile syringes and incubated at 50°C. The enrichment obtained successfully with gelatin was used for further pure culture isolation.

The WB medium with 18 mM lactate was used for pure culture isolation. All cultures were incubated at 70°C starting from this stage of isolation. For pure culture isolation, single colonies were picked up followed by multiple dilution series on WB medium solidified with 0.3% Gelzan^TM^ (Gelrite^®^, Sigma-Aldrich, St. Louis, MO, USA). Culture purity was checked microscopically and by separation and sequencing of partial (585 bp) 16S rRNA gene fragments in a denaturing gradient gel electrophoresis (PCR-DGGE) as previously described (Frank et al., 2016). It was also confirmed by a lack of growth on aerobic Plate Count Agar (per liter: 9.0 g agar, 10 g glucose, 5 g tryptone, and 2.5 g yeast extract) and Anaerobic Agar (Becton Dickinson, Franklin Lakes, NJ, USA) media.

The strain is deposited at the All-Russian collection of microorganisms (VKM) under the accession number VKM B-3066.

### Characterization of Morphology and Physiology

Cell morphology was observed by phase contrast microscopy using an Axio Imager A1 microscope and by transmission electron microscopy (TEM) of ultra-thin sections, as described previously (Ikkert et al., 2013).

All growth experiments were carried out in the modified WB medium with 18 mM lactate as an electron donor. Bicarbonate buffer was excluded from the basal WB media to allow pH modifications and the pH was adjusted with 0.5 M H_2_SO_4_ or 2 M NaOH to the desired experimental pH values. The pH measurements were taken at the incubation temperature, since the pH value maybe a function of temperature (Wiegel, 2011). Growth was analyzed in the pH range from 5.0 to 11.0 at the optimum temperature of 65°C. Growth was tested at different incubation temperatures in the range from 37 to 75°C in 12 mL head-space free test tubes. Growth was determined by microscopic cell counts in triplicate samples. Specific growth rates were calculated from the cell counts during the exponential phase of growth. Further physiological experiments were conducted at 65°C at pH 8.5. The optimum salinity for growth was determined in the range of 0 to 1% NaCl.

Growth was analyzed with the following electron donors: 4.5 mM succinate, 7.5 mM each of formate and malate, 7 mM each of pyruvate and butyrate, 9 mM each of acetate and fumarate, 13.5 mM propionate, 3 mM sucrose, 5 mM each of fructose and glucose, 25 mM ethanol, 17 mM propanol, 13.5 mM butanol, and 11 mM glycerol. Growth on 7.5 mM formate and H_2_ was also tested in presence of 2 mM acetate. Microcrystalline cellulose (100 mg per 12 mL cultivation tube), 1% gelatin, and 1% peptone (Sigma-Aldrich) were also tested as carbon sources and electron donors. Carbohydrate stock solutions were sterilized using polyethersulfone 0.22 μm Millex-GP filter units (Merck Millipore, Darmstadt). If growth was observed, the culture was subcultured at least five times in the presence of each electron donor and acceptor to confirm their utilization. The electron acceptors were 28 mM sodium sulfate, 2 mM and 20 mM sodium sulfite, 20 mM sodium thiosulfate, 10 mM fumarate, 2 mM sodium nitrite, 0.4 mM and 5 mM calcium nitrate, 20 mM Fe(III) citrate, and 2% elemental sulfur. All the presumed acceptors were tested in presence of 18 mM lactate. Growth with nitrite was also checked in presence of 7.5 mM formate (plus 2 mM acetate).

Scanning electron microscopy with energy-dispersive spectroscopic analysis (SEM-EDS) and X-ray diffraction (XRD) were used to check for cellulose formation by strain N1 (de Oliveira et al., 2011). Cells were collected at the end of stationary growth by centrifugation (16,800 g for 10 min) and air-dried. SEM-EDS was performed using a Philips SEM 515 scanning electron microscope as described previously (Ikkert et al., 2013). The presence of crystalline cellulose was analyzed by XRD using a Shimadzu XRD 6000 diffractometer with CuKa radiation (Ikkert et al., 2013). Mineral identification was computer-assisted with the Crystallographica-Search Match and the database PDF-2.

### DNA Isolation and Phylogenetic Analysis

Genomic DNA was isolated from the cells in the late exponential growth phase. Cells from 500 mL of liquid culture were harvested by centrifugation at 4,200 *g* for 40 min and washed with 1× phosphate-buffered saline (PBS), pH 7.4. The cells were lysed with 0.6% sodium dodecyl sulfate and DNA was isolated using phenol-chloroform extraction and precipitated with ethanol as described by Green and Sambrook (2012).

The 16S rRNA gene was amplified using the primer pair 27F and 1492R (Lane, 1991). Phylogenetic analysis of the 16S gene sequences was conducted using the Neighbor-Joining method (Saitou and Nei, 1987) in MEGA6 software (Tamura et al., 2013).

### Genome Sequencing, Assembly, and Analysis

*Thermodesulfovibrio* sp. N1 genomic DNA was sequenced with a Roche Genome Sequencer (GS FLX), using the Titanium XL+ protocol for a shotgun genome library. About 50 Mb of sequences with an average read length of 464 nt were generated and *de novo* assembled into contigs using Newbler Assembler version 2.9 (454 Life Sciences, Branford, CT, USA). The draft genome of *Thermodesulfovibrio* sp. N1 consists of 116 contigs longer than 500 bp, with a total length of 1,915,225 bp.

Gene search and annotation were performed for all contigs longer than 500 bp, using the RAST server (Brettin et al., 2015), followed by manual correction by searching the National Center for Biotechnology Information (NCBI) databases. Signal peptides were predicted using Signal P v.4.1 for Gram-negative bacteria^1^. The N-terminal twin-arginine translocation (Tat) signal peptides were predicted using PRED-TAT^2^ and the transmembrane helices with TMHMM Server v. 2.0^3^.

The values of DNA–DNA hybridization *in silico* were calculated using GGDC 2 (Meier-Kolthoff et al., 2013), available at http://ggdc.dsmz.de/.

### Nucleotide Sequence Accession Number

The annotated genome sequence of *Thermodesulfovibrio* sp. N1 has been deposited in the GenBank database under accession no. NZ_MAVV00000000.

## Results and Discussion

### Source Water Characteristics

The physicochemical parameters of the water measured in August 2013 are shown in **Table 1**. The water was slightly alkaline with pH ranging from 7.9 to 8.2 and the temperature from 40.2 to 44.8°C. The groundwater from the borehole is highly reducing (anaerobic), with sulfate and nitrate being undetectable (<5 and <3 mg L^-1^, respectively). This observation is supported by a very low redox potential of down to -336 mV, the presence of dissolved H_2_S at 0.64 ± 0.35 mg L^-1^, and the presence of methane in the exsolved gases from the borehole.

**Table 1:** Physical and chemical characteristics of the source water at borehole 1-R.

Parameter	Unit	Value
pH		8.21
Eh	mV	-336
T	°C	44.8
t-alkalinity	meq L^-1^	4.7
Hydrogen sulfide (H_2_S)^∗^	mg L^-1^	0.64 ± 0.35
*Major anions determined by ion chromatography*		
Cl^-^	mg L^-1^	843
SO_4_^2-^	mg L^-1^	<5
NO_3_^-^	mg L^-1^	<3
Br^-^	mg L^-1^	3.04
F^-^	mg L^-1^	11.5
*Cations and other elements determined by ICP-MS*		
Na	mg L^-1^	656
Mg	mg L^-1^	0.14
Ca	mg L^-1^	9.4
K	mg L^-1^	3.14
Mn	μg L^-1^	8.3
Fe	μg L^-1^	97
Sr	mg L^-1^	1.00
Ba	μg L^-1^	227
Li	μg L^-1^	46
Al	μg L^-1^	14
Si	mg L^-1^	12.9
P	mg L^-1^	<0.01
S	mg L^-1^	2
B	mg L^-1^	2.27

*^∗^Mean ± standard deviation.*

The groundwater ionic composition was dominated by Na^+^ (656 mg L^-1^) and Cl^-^ (843 mg L^-1^), with a modest component (4.7 meq L^-1^) of bicarbonate alkalinity. The concentrations of dissolved Fe and Mn were modest at about 100 and 8 μg L^-1^ respectively, presumably being suppressed by the high pH of the water and possible precipitation in the form of sulfides.

Ca and Mg occur at very low concentrations in the water, their solubility presumably being suppressed by calcite/dolomite saturation in a high-pH, bicarbonate-rich environment. Bromide was at just over 3 mg L^-1^ in the water, yielding a chloride:bromide mass ratio of about 270. This is very close to seawater (290) and suggests that the aquifer salinity may have its origin in relict ocean water.

The low concentrations of As (0.5 μg L^-1^) and of most of heavy metals reflect their low solubility in high pH, sulfide-rich environments. Tungsten occurred at around 4 μg L^-1^: this element is known to accumulate in high pH groundwater (Frengstad et al., 2001).

The stable isotopic signature (^18^O and ^2^H) suggests that the water is essentially derived from meteoric recharge, with the isotopic signature slightly modified by ^18^O exchange with the aquifer matrix in a mildly geothermal environment or possibly by admixture with a minor component of connate marine water (Banks et al., 2014).

### Pure Culture Isolation and Characterization

Sulfidogenic enrichment was set up by inoculation of gelatin-containing WB medium with groundwater directly from the borehole wellhead. Growth appeared as blackening in the medium due to H_2_S production after 3 weeks of incubation at 50°C and pH 7.5. Microscopic observations revealed different morphotypes, dominated by various rods, with a minority of vibrio-shaped cells. The vibrios became dominant when the culture was incubated with lactate at 70°C. The isolate, designated strain N1, was purified by repeated colony isolation followed by serial dilution. The culture purity was confirmed by microscopic observations and absence of growth on Plate Count Agar (aerobic cultivation) and Anaerobic Agar (anaerobic cultivation). The PCR-DGGE analysis revealed only one phylotype (data are not shown).

**FIGURE 1:**
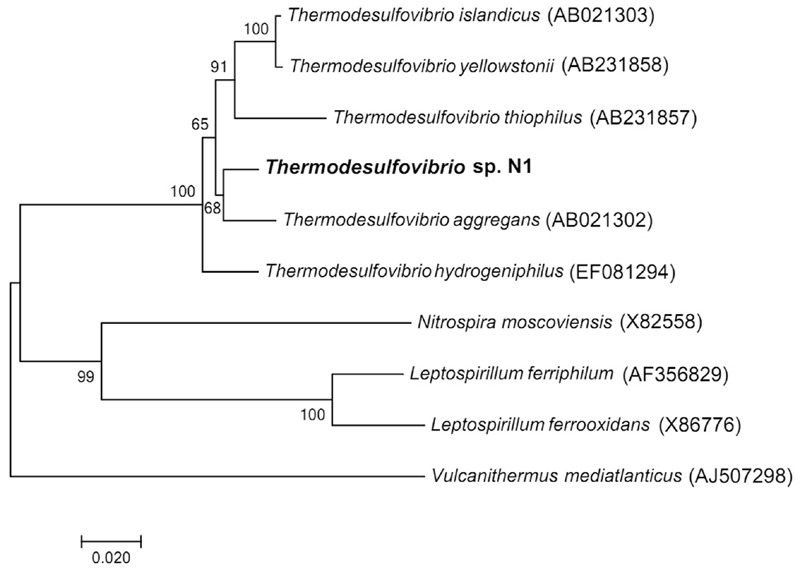
**Neighbor-joining 16S rRNA gene tree, showing the phylogenetic position of strain N1.** The scale bar represents an estimated 2% sequence divergence. Numbers at nodes represent the bootstrap support values. Full-size 16S rRNA gene of strain N1 identified in the genome was used to build the tree. *Vulcanithermus mediatlanticus* was used as an outgroup.

The nearly full sequence (1,386 bp) of the 16S rRNA gene of strain N1 was amplified by PCR. Phylogenetic analysis places strain N1 within phylum *Nitrospirae*, genus *Thermodesulfovibrio*. The closest relative of strain N1 was *Thermodesulfovibrio aggregans*, with a 16S RNA sequence similarity of 97% (**Figure 1**).

**FIGURE 2:**
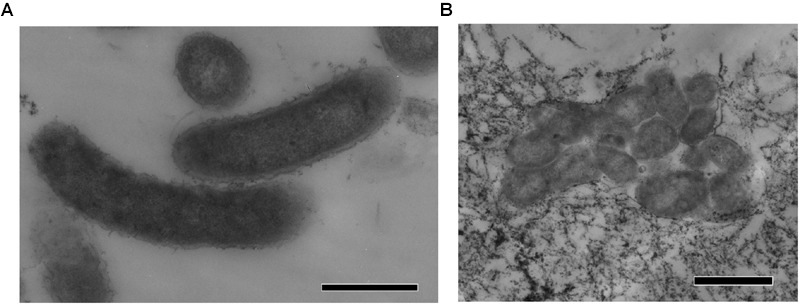
**TEM micrographs of thin sections of *Thermodesulfovibrio* sp. N1. (A)** Free-growing cells. **(B)** Group of cells forming aggregates. The scale bars are 0.5 μm **(A)** and 1 μm **(B)**.

**FIGURE 3:**
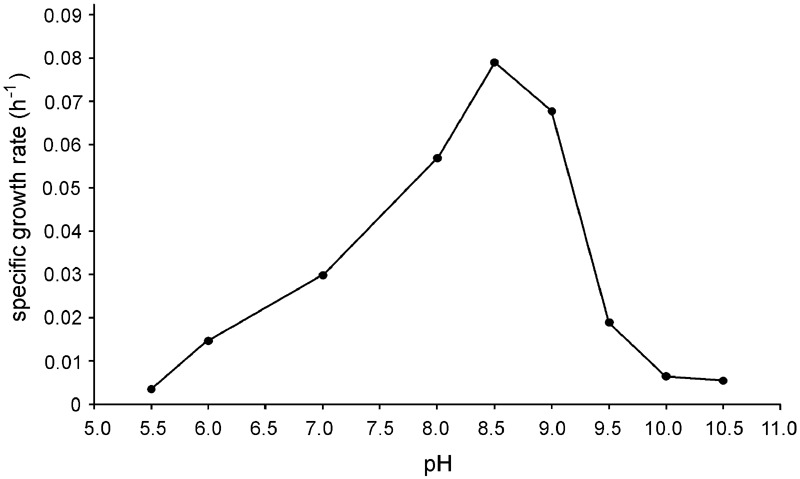
**Effect of pH on the specific growth rate of *Thermodesulfovibrio* sp. N1.** Cultures were grown at different pH values with lactate and sulfate at 65°C.

**FIGURE 4:**
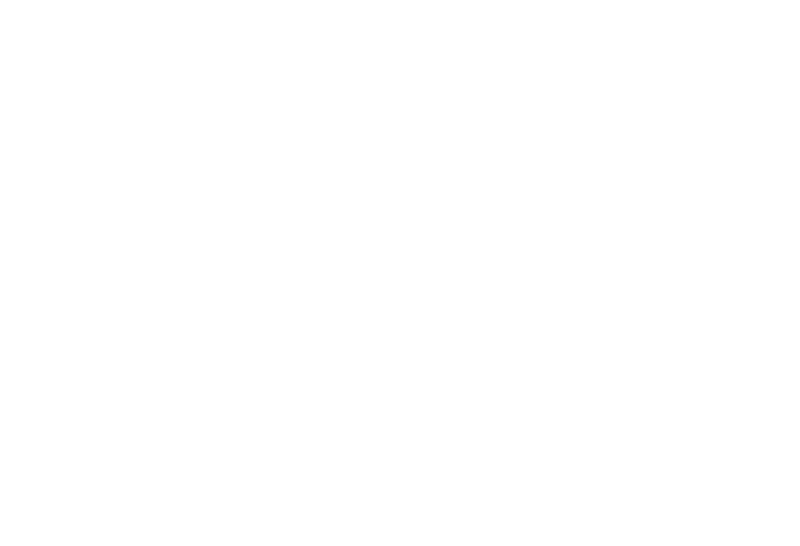
**An overview of the metabolism of *Thermodesulfovibrio* sp. N1 reconstructed from its genome.** Enzyme abbreviations: LDH, lactate dehydrogenase; POR, pyruvate ferredoxin oxidoreductase; ACS, acetyl-CoA synthetase; Tsr, thiosulfate reductase (subunits A, B, and C); Dsr, dissimilatory sulfite reductase; Apr, adenosine-5′-phosphosulfate reductase; QmoABC, adenylylsulfate reductase-associated electron transfer complex; Sat, sulfate adenylyltransferase; PPase, pyrophosphatase; FdhAB, periplasmic formate dehydrogenase; Hmc, membrane-linked Hmc complex; Nrf, cytochrome *c* nitrite reductase; Nuo, membrane-linked complex comprising subunits NuoA, B, C, D, H, I, J, K, L, M, and N of NADH-ubiquinone oxidoreductase; HdrABC, heterodisulfide reductase subunits A, B, and C; MvhADG, methyl viologen-reducing hydrogenase subunits A, D, and G; Hase 1, periplasmic group 1 [NiFe] uptake hydrogenase; Hase 3, cytoplasmic group 3b [NiFe] bidirectional hydrogenase; Hyf, membrane-linked group 4 [NiFe] hydrogenase; PsrAB, subunits A and B of Psr/Psh family oxidoreductase; ATP, F_1_F_0_ ATP synthase; Mnh, a multisubunit Na^+^/H^+^ antiporter of the Mnh family (MnhBCDEFG); BcsABZC, cellulose synthase. Other abbreviations: ox/red, oxidized and reduced forms; cyt, cytochrome *c*; Pi, phosphate; PPi, pyrophosphate; CoA, coenzyme A; OM, outer membrane; CM, cytoplasmic membrane.

Strain N1 is a motile vibrio, 1.5–2.0 μm long and 0.4 μm wide (**Figure 2**). Cells do not produce spores and have a Gram-negative cell wall with an outer membrane. Cells tend to form aggregates and produce an extracellular matrix when grown in biofilms. The presence of cellulose could not be verified in an air-dried cell pellet by SEM-EDS and XRD. The strain is an obligate anaerobe and grows in the temperature range of 45–74°C with an optimum at 65°C. The optimal NaCl concentration was 0.1% and growth ceased at concentrations exceeding 0.6%. Strain N1 could grow in lactate medium at 65°C at initial pH range of 5.5–10.5 (**Figure 3**). The pure culture was isolated on the WB medium with carbonate buffer at pH 7.5, but the growth experiments showed that strain N1 grew faster when carbonate buffer was excluded and pH adjusted to 8.5 with 2 M NaOH. We did not add any buffers to keep alkaline pH in further experiments, but substantial pH changes were not observed in the culture growing at initial pH 8.6. The pH varied slightly during growth and reached 8.7 at the stationary phase (Supplementary Figure S2). The maximum specific growth rate of 0.08 h^-1^ was observed at pH 8.5 (**Figure 4**). Thus, strain N1 can be considered to be a facultatively alkaliphilic bacterium, with an alkaline pH optimum but capable of growing at circumneutral pH (Presis et al., 2015).

Strain N1 used lactate and pyruvate as electron donors for sulfate reduction. The strain grew slowly with formate or H_2_ only when 2 mM acetate was added to the medium as a carbon source. Other organic acids (acetate, malate, succinate, fumarate, propionate, and butyrate), alcohols (ethanol, butanol, propanol, and glycerol), gelatin, peptone, glucose, fructose, and sucrose were not utilized by strain N1 as electron donors. A limited number of substrates is a characteristic trait of all *Thermodesulfovibrio* isolates. Besides sulfate, strain N1 could also use sulfite, thiosulfate and Fe(III) as electron acceptors. However, growth with Fe(III) as electron acceptor was slow. Nitrate, nitrite, fumarate, and elemental sulfur were not used as electron acceptors with lactate as electron donor. Nitrite did not support growth in presence of formate and acetate.

### General Genome Properties

Assembly of the draft genome of strain N1 yielded 116 contigs longer than 500 bp with N50 contig length of 31,594 bp. The genome of *Thermodesulfovibrio* sp. N1, estimated as the total length of all contigs, is about 1.93 Mbp, with a GC content of 34.4%. The size of the genome is comparable to that of other *Thermodesulfovibrio* spp. (1.87–2.05 Mbp). A single 16S–23S rRNA operon, distantly located 5S rRNA gene, and 46 tRNA genes were identified. Gene calling and annotation of the genome predicted 2,048 potential protein-coding genes of which 1,520 (74%) can be functionally assigned. Analysis of the conserved single-copy marker genes using CheckM (Parks et al., 2015) estimated the completeness of the assembly as 99.0%.

The degree of *in silico* DNA–DNA hybridization between strain N1 and other *Thermodesulfovibrio* with known genome sequences (*T. yellowstonii* DSM 11347, *T. aggregans* JCM 13213, *T*. *islandicus* DSM 12570, and *T*. *thiophilus* DSM 17215) was estimated to be in the range 19–21%, indicating that strain N1 represents a novel species of *Thermodesulfovibrio*.

### Metabolic Pathways

*Thermodesulfovibrio* sp. N1 is a sulfate-reducing bacterium growing on a limited number of organic substrates, with lactate and pyruvate identified in this work. Consistent with the inability of strain N1 to grow on simple sugars and polysaccharides, secreted glycoside hydrolases were not found and only a few intracellular enzymes involved in the metabolism of sugars were present. The Embden–Meyerhof glycolytic pathway probably operates in the direction of gluconeogenesis, as indicated by the presence of enzymes specifically performing the reverse reactions: phosphoenolpyruvate synthase (THER_1173) and fructose-1,6-bisphosphatase (THER_0969).

Pyruvate could be reversibly decarboxylated to acetyl-CoA by pyruvate:ferredoxin oxidoreductase (THER_1537- THER_1540). Further conversion of acetyl-CoA to acetate with the production of ATP is performed by acetyl-CoA synthetase (THER_1590). Acetate permease is encoded at the same genome region (THER_1591). Oxidation of lactate to pyruvate is probably enabled by putative lactate dehydrogenases (LDH) belonging to the CCG family, encoded by the gene cluster THER_0621- THER_0617. The cluster comprises lactate permease, putative glycolate oxidase subunit GlcD, Fe-S oxidoreductase with a 4Fe-4S dicluster domain and two CCG domains, an L-lactate utilization protein LutC, and a LutB protein containing DUF162, a 4Fe-4S dicluster, and two CCG domains. Similar loci were previously identified in the genomes of various sulfate-reducing bacteria, particularly in *Desulfovibrio vulgaris* (Pereira et al., 2007). It was suggested that this LDH is bound to the membrane via interaction with lactate permease in *D. vulgaris* (Pereira et al., 2007).

The tricarboxylic acid (TCA) cycle in *Thermodesulfovibrio* sp. N1 is incomplete, lacking citrate synthase and succinyl-CoA synthetase. This finding is consistent with the observed inability of strain N1 to oxidize organic substrates completely. For example, growth of strain N1 with formate was only possible in the presence of acetate as an auxiliary carbon source, consistent with the absence of known pathways for autotrophic C1 fixation. In particular, the Wood–Ljundahl (reductive acetyl-CoA) pathway, frequently used by autotrophic sulfate reducers, is incomplete, lacking carbon monoxide dehydrogenase/acetyl CoA synthase.

The genome of *Thermodesulfovibrio* sp. N1 contains all genes necessary for dissimilatory sulfate reduction (Rabus et al., 2007): sulfate adenylyltransferase (THER_0839), soluble manganese-dependent inorganic pyrophosphatase (THER_1978), adenylylsulfate reductase AprAB (THER_0836/THER_0837), dissimilatory sulfite reductase DsrAB (THER_1044/THER_1045) and distantly encoded DsrC subunit (THER_0471). A three-gene operon encoding the subunits QmoA (THER_0835), QmoB (THER_0834), and QmoC (THER_0833) of the adenylylsulfate reductase-associated electron transfer complex QmoABC is located downstream of the adenylylsulfate reductase genes. The presence of transmembrane helices in QmoC indicates that this subunit links the QmoABC complex to the cytoplasmic membrane, thus enabling electron transfer from the quinone pool to AprAB (Venceslau et al., 2010). Another linkage of sulfate-reduction enzymes to the membrane is enabled by the sulfite reductase-associated electron transfer complex DsrMKJOP (THER_0473–THER_0477), with subunits DsrM and DsrP containing transmembrane domains.

Four hydrogenases of the [NiFe]-family and one formate dehydrogenase are encoded by the *Thermodesulfovibrio* sp. N1 genome. Consistently, like all other *Thermodesulfovibrio* species studied so far, *Thermodesulfovibrio* sp. N1 can grow with H_2_ as an energy source in the presence of acetate as a carbon source. The first hydrogenase is encoded by eight-gene operon (THER_0390- THER_0379), with subunits similar to HyfGHDBCEFI of *Escherichia coli* hydrogenase 4. These group 4 membrane-linked multimeric enzymes catalyze the reduction of H^+^ with ferredoxin coupled to energy conservation in the form of a transmembrane proton gradient (Vignais and Billoud, 2007).

The second group 1 [NiFe] uptake hydrogenase, comprising small (THER_0905) and large (THER_0904) subunits, is probably located in the periplasm, as evidenced by the presence of a Tat motif at the N terminus of its small subunit. Usually, these enzymes oxidize H_2_, donating the electrons to the quinone pool via the third cytochrome *b* subunits linking them to the cytoplasmic membrane. The third subunit is, however, absent in this hydrogenase. The electron transfer from this soluble periplasmic complex to the cytoplasmic membrane may be facilitated by a pool of *c*-type cytochromes present in the periplasm (Pereira et al., 1998; Matias et al., 2005). The product of THER_1799, encoding class III cytochrome *c* carrying an N-terminal signal peptide, could perform this function. This electron transport pathway probably ends at the membrane-linked Hmc complex (Rossi et al., 1993), encoded by genes THER_1440- THER_1444. Hmc comprises ferredoxin, with a 4Fe-4S dicluster domain and a CCG domain (THER_1444), periplasmic 4Fe-4S ferredoxin (THER_1441), an integral membrane protein of the NrfD family (THER_1440) and a large periplasmic 16-heme cytochrome *c* (THER_1443). This cytochrome, HmcA, can accept electrons from periplasmic cytochromes.

An operon of genes THER_1123–THER_1129 codes for cytoplasmic hydrogen:heterodisulfide oxidoreductase, consisting of CoB-CoM heterodisulfide reductase (HdrACB) and methyl viologen-reducing hydrogenase (MvhDGA). This complex was proposed to catalyze the endergonic reduction of ferredoxin and the exergonic reduction of heterodisulfide, coupled to H_2_ oxidation by electron bifurcation involving HdrA (Thauer et al., 2008).

In addition, the *Thermodesulfovibrio* sp. N1 genome encodes four-subunit soluble cytoplasmic hydrogenase (THER_1862–THER_1865), assigned to group 3b. These hydrogenases are bidirectional and can re-oxidize the cofactors by using protons as electron acceptors (Vignais and Billoud, 2007). The presence of an NADPH-binding motif in the THER_1864 protein suggests that this hydrogenase can use NADPH in hydrogen turnover reactions.

The presence of formate dehydrogenase explains the observed ability of *Thermodesulfovibrio* sp. N1 to use formate as an electron donor. This enzyme is encoded by genes THER_1543 (catalytic subunit A) and THER_1541 (Fe-S containing subunit B). The presence of an N-terminal Tat signal peptide in FdhA suggests that it is located in the periplasm. Like the periplasmic uptake hydrogenase, formate dehydrogenase lacks a membrane subunit, and electron transfer to the membrane is probably performed via the periplasmic cytochromes and Hmc complex.

Genome ànalysis of *Thermodesulfovibrio* sp. N1 revealed several other membrane-linked oxidoreductases that can contribute to the generation of transmembrane ion gradient and/or the use of alternative electron acceptors to sulfate. Two putative complexes similar to the bacterial NADH:quinone oxidoreductase are encoded by gene clusters THER_0569–THER_0582 and THER_1527–THER_1536. Both comprise the subunits similar to NuoA, B, C, D, H, I, J, K, L, M, and N, while the genes for the subunits NuoEFG that form the NADH dehydrogenase module are missing, indicating that NADH is likely not an electron donor. The first cluster is linked to genes coding for two subunits of CISM oxidoreductases of the Psr/Psh family (reviewed by Rothery et al., 2008): the molybdopterin-binding catalytic subunit A (THER_0567) and the iron-sulfur electron transfer subunit B (THER_0568). Phylogenetic analysis of the catalytic subunit classifies it as thiosulfate or polysulfide reductase (Supplementary Figure S3). It is possible that such an arrangement indicates coupling of transmembrane proton transfer, performed by the core subunits of NADH:quinone oxidoreductase, with the oxidation or reduction of sulfur compounds. Genes for the second NuoABCDHIJKLMN complex are located immediately downstream of the pyruvate:ferredoxin oxidoreductase (THER_1537–THER_1540) and this arrangement is conserved also in *T. yellowstonii*. Oxidation of pyruvate produces reduced ferredoxin that could provide electrons to this oxidoreductase.

Genes THER_0551–THER_0553 encode molybdopterin family oxidoreductase consisting of all three subunits: A, B and membrane subunit C of the NrfD family. The catalytic A subunit was predicted to contain an N-terminal Tat signal peptide and is phylogenetically related to thiosulfate or polysulfide reductases (Supplementary Figure S3). The presence of putative thiosulfate reductase, capable of producing sulfide and sulfite from thiosulfate, explains the ability of *Thermodesulfovibrio* sp. N1 to use thiosulfate as an electron acceptor. Thiosulfate reduction also could be performed by the Dsr complex (Venceslau et al., 2014).

In spite of the observed inability of *Thermodesulfovibrio* sp. N1 to grow by nitrite reduction, its genome contains cytochrome *c* nitrite reductase (Rodrigues et al., 2006), comprising large NrfA (THER_1503) and small NrfH (THER_1504) subunits with five and four hemes, respectively. The presence of an N-terminal signal peptide in the large subunit suggests that this complex faces the periplasmic side of the membrane. The physiological role of nitrite reductase could be detoxification of nitrite, which is known as an inhibitor of sulfate-reducing organisms (Greene et al., 2003). A similar function in detoxification of oxygen could be assigned to the cytochrome *bd* ubiquinol oxidase (THER_1407 and THER_1409).

Overall, the sulfate reduction machinery and related metabolic pathways in *Thermodesulfovibrio* sp. N1 resemble those of *Deltaproteobacteria* rather than of *Firmicutes*. Their characteristic hallmarks are the QmoABC complex linked to the membrane via its C subunit, a full DsrMKJOP module, periplasmic hydrogenases and *c*-type cytochromes (Pereira et al., 2011). An overview of the metabolic pathways, revealed by genome analysis, is shown in **Figure 4**.

### Comparative Genomics of *Thermodesulfovibrio*

To date, no alkaliphilic or acidophilic forms have been detected within genus *Thermodesulfovibrio*. The revealed alkaliphilic growth character of *Thermodesulfovibrio* sp. N1 is consistent with the slightly alkaline deep groundwater environment. Strain N1 is the first facultatively alkaliphilic representative of the genus to be characterized.

Comparative analysis of the *Thermodesulfovibrio* sp. N1 genome and the four other sequenced genomes of *Thermodesulfovibrio* species (*T. aggregans, T. thiophilus, T. islandicus*, and *T. yellowstonii*) revealed a very low genetic diversity within this genus (**Table 2**). Most of the species-specific genes were associated with integrated mobile and prophage-like elements or annotated as “hypothetical proteins.” In particular, all four *Thermodesulfovibrio* genomes contain the sulfate reduction pathway (*sat, aprAB, dsrABC, qmoABC*, and *dsrMKJOP*), four types of hydrogenases, periplasmic formate dehydrogenase, an Hmc complex and periplasmic cytochromes, NADH:quinone oxidoreductase-like complexes lacking NADH dehydrogenase module, similar molybdopterin oxidoreductases and periplasmic nitrite reductase. All genomes encode an incomplete TCA cycle, while none encodes pathways for autotrophic C1 fixation. However, several specific traits related to metabolic capabilities and lifestyles were uncovered.

**Table 2:** Comparative phenotypic and genomic features of *Thermodesulfovibrio* species.

Property	*T. yellowstonii* (DSM 11347)	*T. islandicus* R1Ha3 (DSM 12570)	*T. àggregans* TGE-P1 (DSM 17283)	*T. thiophilus* TDV (DSM 17215)	Strain N1
**Phenotypic features**
Isolation source	Hydrothermal vent water	Terrestrial hot spring	Thermophilic anaerobic sludge	Thermophilic anaerobic sludge	Subsurface thermal aquifer
Temperature range for growth (optimum), aaaC	40–70 (65)	45–70 (65)	45–70 (60)	45–60(55)	45–74
pH range for growth (optimum)	6.5–7.7 (6.8–7.0)	(6.8–7.0)	6.0–8.5 (6.5–7.0)	6.0–8.5 (7.0–7.5)	5.5–10.5 (8.5–9.0)
**Electron donors:**
Lactate	+	+	+	+	+
Pyruvate	+	+	+	+	+
Formate^∗^	+	+	+	+	+
H_2_^∗^	+	+	+	+	+
Acetate	-	-	-	-	-
Ethanol	-	nd	-	-	-
**Electron acceptors:**
Sulfate	+	+	+	+	+
Thiosulfate	+	+	+	+	+
Sulfite	+	–	–	+	+
Elemental sulfur	-	-	-	-	-
Nitrate	-	+	-	-	-
Fe(III)	+	nd	+	+	+
*Genomic properties*
Genome size (Mb)	2.00	2.04	1.97	1.87	1.93
GC (%)	34.1	34.3	34.8	34.4	34.4
Number of protein-coding genes	2,028	2,036	2,097	1,864	2,048
Sulfate reduction pathway: *sat, aprAB, qmoABC, dsrAB, dsrC, dsrMKJGOP*	+	+	+	+	+
**Hydrogenases**
[NiFe] group 1	+	+	+	+	+
[NiFe] group 3b	+	+	+	+	+
Methyl viologen-reducing hydrogenase	+	+	+	+	+
[NiFe] group 4	+	+	+	+	+
Fe only	+	+	-	-	-
Nitrogenase	+	+	+	-	-
Nitrate reductase	-	+	-	-	-
Na^+^/H^+^ antiporter of the Mnh family	-	-	-	-	+
Cellulose synthase genes	-	-	+	-	+

*^∗^ With 2 mM acetate.*

*nd, no data available.*

Consistent with its alkaliphilic lifestyle, the genome of *Thermodesulfovibrio* sp. N1 encodes a multisubunit Na^+^/H^+^ antiporter of the Mnh family (THER_0672–THER_0680). Such antiporters play a major role in Na^+^ extrusion and pH adaptation (Ito et al., 1999). None of the other available *Thermodesulfovibrio* genomes contains this multisubunit Na^+^/H^+^ antiporter.

Present in the genomes of *Thermodesulfovibrio* sp. N1 and *T. aggregans*, the cellulose synthase operon encodes the main catalytic glycosyltransferase subunit BcsA (THER_1683), the periplasmic cyclic di-GMP binding subunit BcsB (THER_1684), the endo-1,4-beta-D-glucanase BscZ (THER_1685) and the periplasmic subunit BcsC (THER_1686). Upstream of the *bcsABZC* operon is the gene THER_1683, encoding a putative regulatory protein with PAS and GGDEF domains. The structure of the *bcs* operon suggests that it could be assigned to type 3, as proposed by Römling and Galperin (2015). Besides their counterparts in *T. aggregans*, the *bcs* genes have no close homologs in other *Nitrospirae* and have likely been acquired laterally. In *Thermodesulfovibrio* sp. N1 and *T. aggregans*, the cellulose synthase is probably responsible for the synthesis of an extracellular polysaccharide matrix. This can explain the observed ability of *T. aggregans* TGE-P1^T^ to form dense cell aggregates (flocs) in a liquid medium (Sekiguchi et al., 2008). Likewise, cellular aggregation was observed when *Thermodesulfovibrio* sp. N1 was grown in liquid WB medium (**Figure 2B**). This trait may be important in deep subsurface aquifer systems where bacteria may form biofilms in organic-rich niches. However, cellulose presence has not been detected in air-dried strain N1 cell pellets by SEM-EDS and XRD.

The genomes of *T. yellowstonii* and *T. islandicus* encode an additional hydrogenase of the Fe-only type. This four-subunit enzyme (THEYE_A1725–THEYE_A1728 in *T. yellowstonii*) is probably located in the periplasm, since its small subunit contains an N-terminal Tat signal sequence.

*T. yellowstonii, T. aggregans*, and *T. islandicus* contain all genes necessary for nitrogen fixation, including the molybdenum-iron nitrogenase and accessory proteins, and they can fix N_2_ for growth. However, diazotrophic growth has not been reported for any *Thermodesulfovibrio* species. Unlike other members of the genus, *T. islandicus* can use nitrate as an electron acceptor (Sonne-Hansen and Ahring, 1999). Consistently, two genes were found in the genome, encoding periplasmic NapAB-type nitrate reductase (H530_RS0102880 and H530_RS0102885). Nitrite, produced from nitrate by this enzyme, could be further reduced to ammonium by nitrite reductase, found in all five genomes.

Overall, the genome sequences of *Thermodesulfovibrio* sp. N1 provide novel information about the metabolic pathways in this microorganism thriving in the deep subsurface aquifer environment. Molecular analysis, by pyrosequencing of 16S rRNA gene fragments, of the microbial community of the deep subsurface thermal water, sampled via borehole 1-R at Byelii Yar, revealed that *Thermodesulfovibrio* sp. N1 accounted for about 2.4% of the 16S rRNA gene reads (our unpublished data). Known sulfate-reducing bacteria comprised about 30% of the community and were mostly represented by chemolithoautotrophic groups, with heterotrophic bacteria and uncultured lineages accounting for the rest of the community. Being unable to grow without organic carbon sources, *Thermodesulfovibrio* depends on fermentation products produced by the heterotrophic part of the community. The abundant population of heterotrophic bacteria in the thermal water provides such substrates for *Thermodesulfovibrio*, making them competitive with chemolithoautotrophs.

## Author Contributions

OK, YF, and NR designed the research project and wrote the paper; YF, VK, AL, AM, ES, and MA performed the research; VK, DB, AB, OK, and NR analyzed the data.

## Conflict of Interest Statement

The authors declare that the research was conducted in the absence of any commercial or financial relationships that could be construed as a potential conflict of interest.

## Footnotes

1 http://www.cbs.dtu.dk/services/SignalP/

2 http://www.compgen.org/tools/PRED-TAT/

3 http://www.cbs.dtu.dk/services/TMHMM/
