# Supplementary material for: Characterization and Genome Analysis of the First Facultatively Alkaliphilic Thermodesulfovibrio Isolated from the Deep Terrestrial Subsurface
Source: Front Microbiol. 2016 Dec 19;7:2000. doi: 10.3389/fmicb.2016.02000 (PMC5165239; doi:10.3389/fmicb.2016.02000)
Supplement: Supplementary file 1 [file Image_1.pdf]

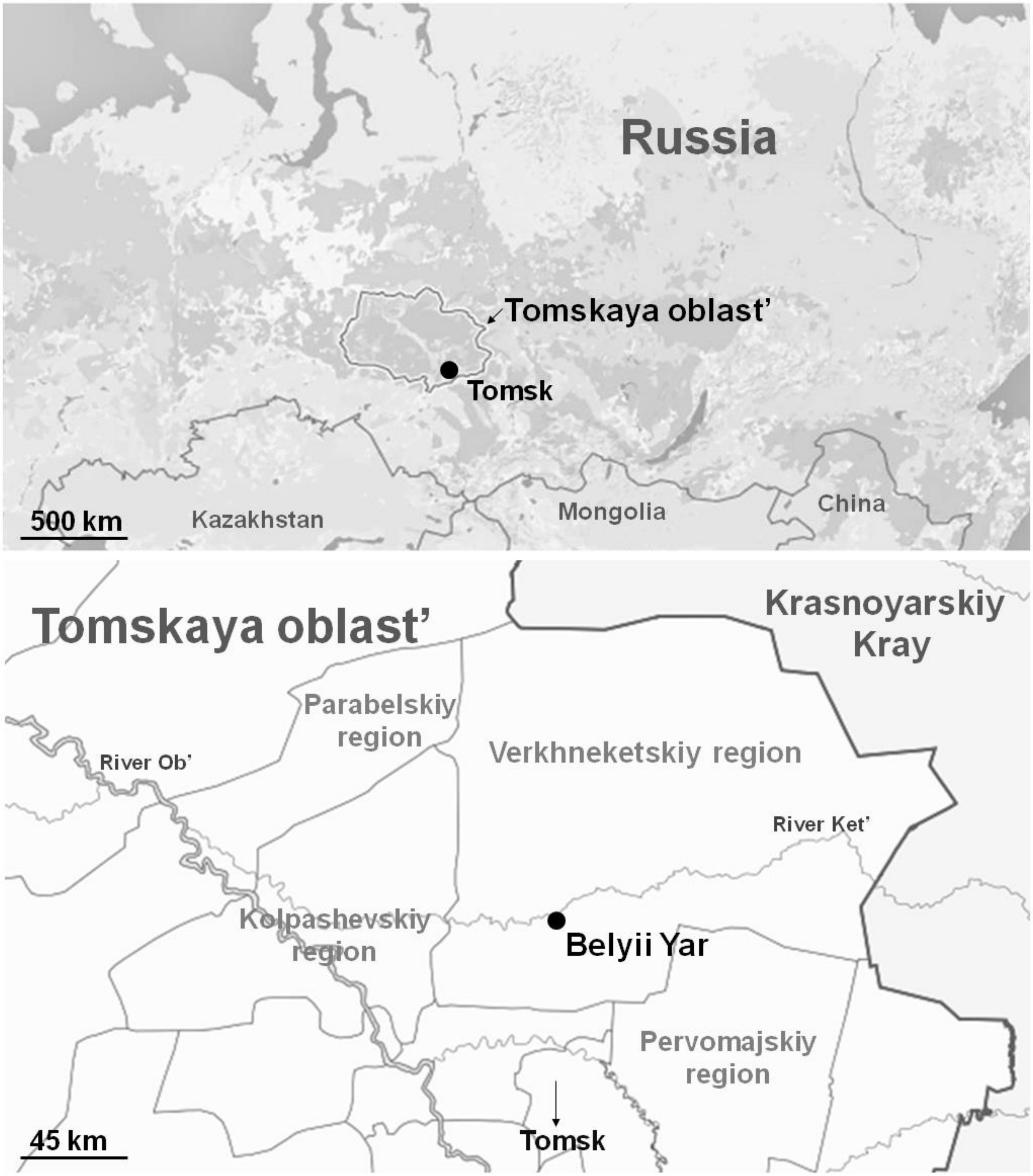

Figure S1. Location map.

The upper map shows the location of Tomskaya oblast' and Tomsk City in Western Siberia, Russia. The lower map shows the location of the studied borehole located in the town of Byelii Yar in Tomskaya oblast'.

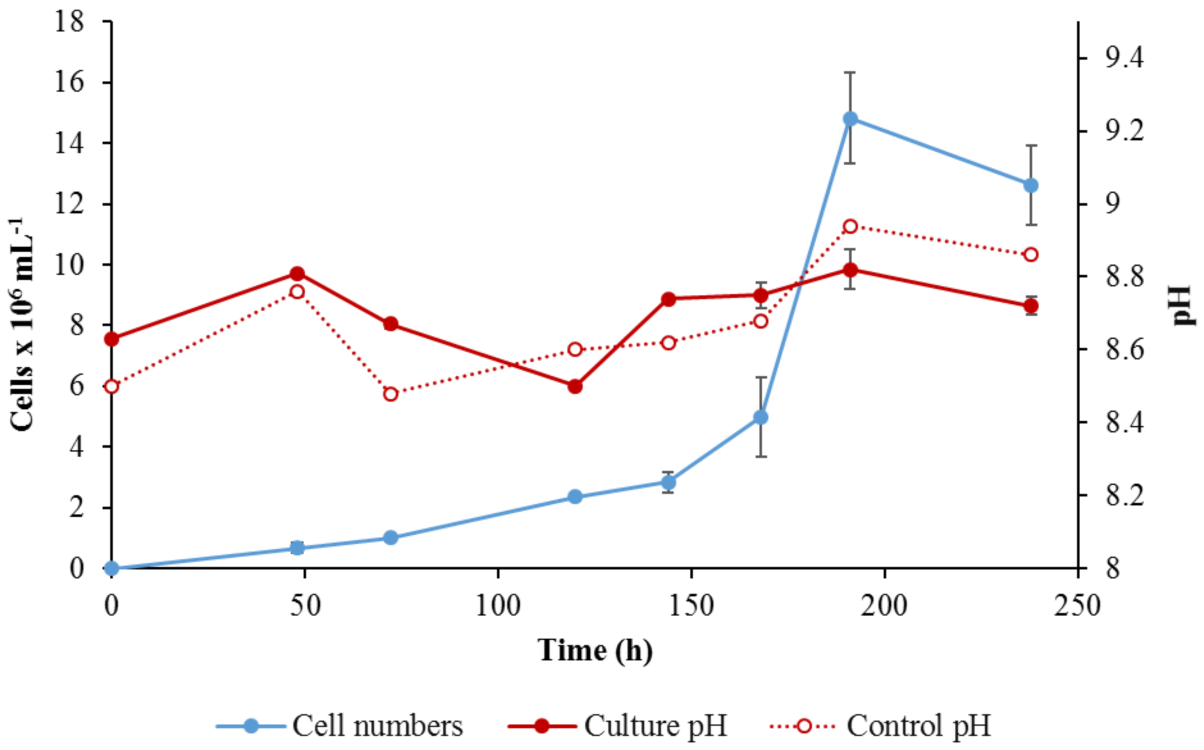

Figure S2. Changes in pH in the control medium without cells and in the culture medium during the growth of *Thermodesulfovibrio* sp. N1.

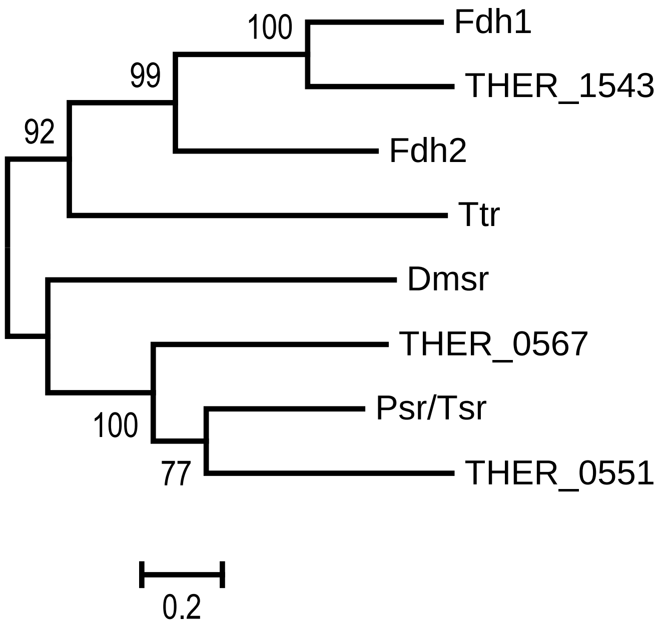

Figure S3. Phylogenetic tree of the catalytic A subunits of molybdopterin oxidoreductases.

For phylogenetic analysis of the catalytic A subunits of molybdopterin oxidoreductases the *Thermodesulfovibrio* sp. N1 proteins THER_0551, THER_0567, and THER_1543 were used along with consensus sequences of the A subunits of tetrathionate reductases (Ttr), formate dehydrogenases (Fdh1 and Fdh2), thiosulfate or polysulfide reductases (Psr), and DMSO reductases (Dmsr), defined in (Yanyushin et al., 2005). Amino acid sequences were aligned using MUSCLE, ambiguously aligned sites were removed using trimAl before the phylogenetic reconstruction. The maximum likelihood phylogenetic tree was computed by PhyML 3.1, using the gamma model of rate heterogeneity (four discrete rate categories, an estimated alpha-parameter) and LG substitution matrix. Numbers at nodes represent the support values estimated by an approximate Bayesian method. The scale bar represents the average number of substitutions per site.

Yanyushin, M.F., del Rosario M.C., Brune, R.E., and Blankenship, R.E. (2005). New class of bacterial membrane oxidoreductases. *Biochemistry* 44, 10037-10045.
